# cGAS-STING: insight on the evolution of a primordial antiviral signaling cassette

**DOI:** 10.12703/r/10-54

**Published:** 2021-06-08

**Authors:** Hua Cai, Jean-Luc Imler

**Affiliations:** 1 Sino-French Hoffmann Institute, School of Basic Medical Science, Guangzhou Medical University, Guangzhou, China; 2 Université de Strasbourg, CNRS UPR9022, Institut de Biologie Moléculaire et Cellulaire, Strasbourg, France

**Keywords:** innate immunity, cGAMP- 2’3’, cGAMP, CBASS, CD-NTase, interferon

## Abstract

Stimulator of interferon genes (STING) functions in the cytosolic DNA-sensing pathway of innate immunity in mammals. It is activated upon binding the cyclic dinucleotide 2′3′-cGAMP, a second messenger produced by the enzyme cyclic guanosine monophosphate–adenosine monophosphate synthase (cGAS), which acts as the receptor for DNA in this pathway, and triggers the expression of interferons and other viral stress-induced genes. The ancient origin of STING in the evolution of animals had been noted, but its primitive function was speculative. We review here recent advances in the remarkable history of cGAS-STING signaling, which establish that cGAS is a member of the family of cGAS/DncV-like nucleotidyltransferases (CD-NTases). In bacteria, CD-NTases synthesize a wide range of cyclic oligonucleotide second messengers in response to bacteriophage infections, which in turn activate a variety of effector proteins to abort phage infection. Among these effectors, some are related to STING, revealing an ancestral function for the cGAS-STING cassette in antiviral host defense. Study of STING signaling in invertebrate animals is consistent with an early acquisition in the history of metazoans of CD-NTase- and STING-encoding genes to counter the universal threat of viruses. In particular, STING-dependent immunity appears to play a previously unsuspected important role in some insects. These discoveries open up interesting perspectives for the use of model organisms to decipher emerging aspects of cGAS-STING biology in mammals, such as the activation of interferon-independent responses or the function and regulation of cGAS in the nucleus.

## Introduction

Innate immunity is the first line of host defense that operates in all animals to counter infections. It relies on families of receptors known as pattern recognition receptors (PRRs) that sense molecular patterns shared by microorganisms but are absent from the host (e.g. bacterial lipopolysaccharides and fungal β-glucans) and trigger the expression of antimicrobial molecules and cytokines to counter the infection and alert the host of an infectious danger^1^. In mammals, innate immunity participates in activation of the B and T lymphocytes mediating adaptive immunity. Among infectious agents, viruses are a serious threat to all living organisms, be they animals, plants, fungi, protists, or even prokaryotes. They offer few targets for recognition, and triggering of antiviral immunity largely relies on sensing of viral RNA or DNA. As a result, all cells are equipped with powerful mechanisms to sense viral nucleic acids and restrict viruses. Some of these mechanisms are sequence specific and rely on RNA guides to specifically neutralize viral nucleic acids, e.g. CRISPR/Cas in prokaryotes or RNA interference (RNAi) in plants, fungi, and some invertebrates like worms and insects^2,3^. In vertebrates, nucleic acid-sensing PRRs, e.g. the Toll-like receptors TLR3 for double stranded (ds)-RNA, TLR7 for single stranded (ss)-RNA, or TLR9 for DNA in the endosomes and the RIG-I like receptors for uncapped and dsRNA in the cytosol, trigger a strong, dedicated transcriptional response to establish antiviral immunity (reviewed in 4,5). Among the genes induced by viruses in vertebrates, type I and type III interferon (IFN) cytokines play a major role in the antiviral response through induction or upregulation of hundreds of IFN-stimulated genes (ISGs)^6^. Importantly, nucleic acid-sensing PRRs have to distinguish between self and viral nucleic acids, and this discrimination is crucial because abnormal induction of IFN can lead to severe autoinflammatory diseases (reviewed in 5,7). Hence, investigation of these receptors and their regulation is of crucial importance.

**Figure 1.**
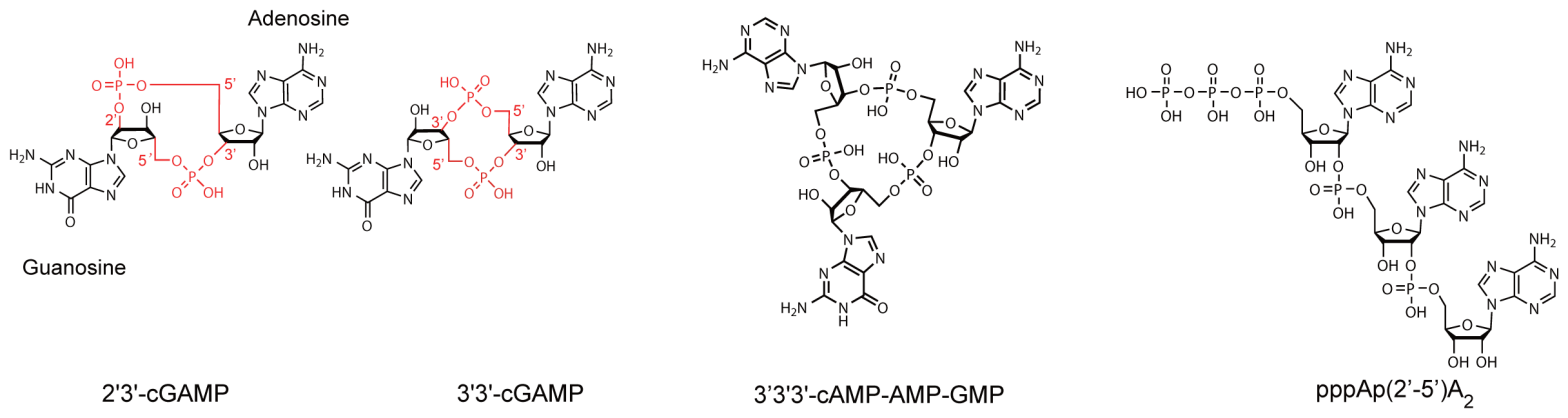
Oligonucleotide second messengers produced by CD-NTases across kingdoms. Cyclic guanosine monophosphate (GMP)–adenosine monophosphate (AMP) synthase (cGAS)/DncV-like nucleotidyltransferases (CD-NTases) synthesize an array of cyclic or linear oligonucleotides connected by 2′–5′ or 3′–5′ bonds. 2′3′-cGAMP and 2′-5′ oligoadenylate—the trinucleotide pppAp(2′–5′)A_2_ is shown—are produced by the mammalian enzymes cGAS and oligoadenylate synthase 1 (OAS1), respectively. 3′3′-cGAMP and 3′3′3′-cAMP-AMP-GMP are produced by the bacterial enzymes DncV (*Vibrio cholerae*) and *Ec*CdnD02 (*Enterobacter cloacae*).

**Figure 2.**
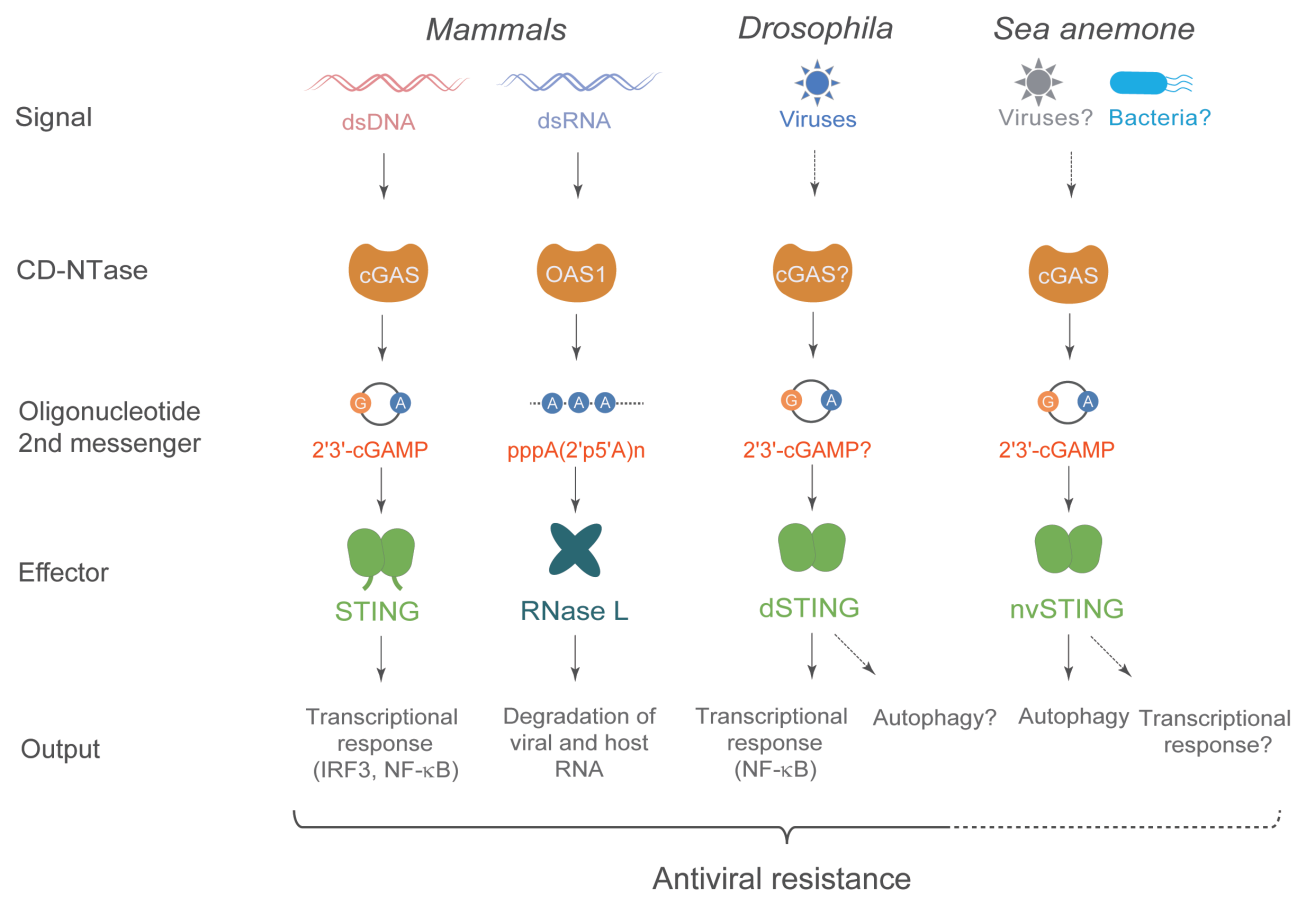
CD-NTase signaling in metazoans. In mammals, members of the cyclic guanosine monophosphate–adenosine monophosphate synthase (cGAS)/DncV-like nucleotidyltransferase (CD-NTase) family are activated by viral nucleic acids to produce second messengers that activate antiviral effector mechanisms. A cGAS-stimulator of interferon genes (STING) cassette is present in the sea anemone *Nematostella vectensis*, but its physiological function is still unclear, although NvSTING can activate autophagy in mammalian cells. In the model insects *Drosophila melanogaster* and *Bombyx mori*, STING participates in antiviral immunity. The CTT extension characteristic of mammalian STING, which mediates docking of TANK-binding kinase 1 (TBK1) and interferon regulatory factor 3 (IRF3), is shown. ds, double stranded; OAS1, oligoadenylate synthase 1.

In mammals, the presence of DNA in the cytosol is sensed by the enzyme cyclic guanosine monophosphate (GMP)–adenosine monophosphate (AMP) synthase (cGAS), which triggers the production of a cyclic dinucleotide (CDN) containing one phosphodiester bond between the 2′-hydroxyl of GMP and the 5′-phosphate of AMP and another between the 3′-hydroxyl of AMP and 5′-phosphate of GMP (2′3′-cGAMP) (Figure 1). Interestingly, cGAS shares closely related structural and enzymatic features with members of the IFN-regulated oligoadenylate synthase (OAS) family, which are encoded by ISGs^8^. The three catalytically active members of the family in humans (OAS1, OAS2, and OAS3) produce a linear non-canonical RNA consisting of 3- to 30-nucleotide-long 2′–5′-linked oligoadenylates. This second messenger activates the latent ribonuclease RNase L, another IFN-regulated gene, to promote the destruction of invading RNAs (Figure 2). The cGAS product 2′3′-cGAMP also acts as a second messenger, binding with nanomolar affinity to a receptor on the membranes of the endoplasmic reticulum: stimulator of IFN genes (STING). STING can also be activated by bacterial cyclic dinucleotides containing two 3′-5′-phosphodiester linkages, such as 3′3′-cGAMP, c-di-GMP, and c-di-AMP, and can therefore activate innate immune responses independently from cGAS upon direct sensing of bacterial products (Figure 1). Upon activation, STING translocates to the membranes of the Golgi apparatus, where it engages the kinase TBK1 through its C-terminal tail (CTT), resulting in phosphorylation of the transcription factor IFN regulatory factor 3 (IRF3) and inducing transcription of the genes encoding type I and III IFN expression (reviewed in 8–10). Of note, STING is an evolutionarily conserved gene, and its presence in animals pre-dates the appearance of IFNs^11^. In particular, the sea anemone *Nematostella vectensis*, which shared a common ancestor with mammals over 600 million years ago, contains a cGAS enzyme producing 2′3′-cGAMP and a STING molecule to which it can bind, raising the question of the ancestral function of these molecules^12–14^ (Figure 2).

Here, we first present recent developments with global relevance in the field of cGAS-STING signaling in mammals. We then discuss the IFN-independent functions of this signaling pathway in invertebrates. Finally, we review new findings revealing that cGAS antiviral signaling has its roots in prokaryotes, where it plays a major role in defense against bacteriophage infections.

## Novel insights on cGAS-STING signaling in mammals

We emphasize here three recent developments in the field of cGAS-STING signaling, which relate to (i) the mechanisms regulating cGAS activity to prevent unwarranted activation of the pathway, (ii) the role of 2′3′-cGAMP as an immunotransmitter, and (iii) the function of STING beyond IFN regulation.

### Regulation of cGAS activity

A central question in the field of cGAS-STING pertains to the mechanisms ensuring that signaling is not induced by self-nucleic acids (reverse transcribed RNA from active retroelements, damaged DNA escaping from the nucleus or stressed mitochondria). This question has important implications for understanding autoinflammatory conditions, e.g. STING-associated vasculopathy with onset in infancy (SAVI), but also neurodegenerative diseases such as Parkinson’s disease, tumor growth, and aging (reviewed in 5,7). Two mechanisms controlling activation by self-DNA have recently emerged. First, DNA induces liquid phase condensation of cGAS, resulting in membrane-less droplets enriched in cGAS and DNA^15,16^. These subcellular condensates function as micro-reactors for highly efficient 2′3′-cGAMP production. Ligand-induced phase separation provides an astute mechanism to define a threshold concentration of DNA to activate the system and avoid reaction to low concentrations of cellular DNA in the cytosol. A second, more recently uncovered, mechanism relates to the regulation of the intracellular localization of cGAS, which does not primarily reside in the cytosol, as initially thought. Indeed, cGAS was reported to associate with the plasma membrane through interaction between its unstructured positively charged N-terminus and negatively charged phosphoinositides^17^. However, an alternative explanation for the regulatory function of this N-terminal region, involving phosphorylation of several serine and threonine residues by mitotic kinases to prevent interaction with chromatin DNA upon nuclear envelope breakdown during mitosis, was recently provided^18^. More surprisingly and somewhat counterintuitively, a large number of cGAS molecules reside in the nucleus, where genomic DNA surrounds them^19,20^. Nuclear cGAS binds tightly to a negatively charged acidic patch formed by the histones H2A and H2B in addition to nucleosomal DNA. This inhibits oligomerization of cGAS, trapping it into an inactive state in the nucleus^21–25^. Barrier-to-autointegration factor 1 (BAF) also participates in the restriction of cGAS activity in the nucleus by displacing transiently bound cGAS molecules from genomic DNA^26^. While future studies will be required to understand the mechanisms controlling the distribution of cGAS in the nucleus versus the cytosol, in particular during infection, this recent discovery provides a tantalizing model for the control of cGAS activity in the absence of infection, with nucleosomes acting as a hallmark of self-DNA. Of note, these results also raise fascinating questions of possible as-yet-unknown nuclear-specific functions of cGAS (e.g. 27,28).

### 2′3′-cGAMP can function as an immunotransmitter

It was realized early on that 2′3′-cGAMP did not function solely as a second messenger in a cell autonomous manner but was able to trigger antiviral immunity in adjacent cells by diffusion through gap junctions^29^ or upon packaging in virions^30,31^. It is now apparent that this CDN also functions as an immunotransmitter released from infected cells into the extracellular space and diffusing to bystander cells, which it enters through either the folate-organic phosphate antiporter SLC19A1 or LRRC8 volume-regulated anion channels (VRACs)^32–35^. These transporters probably operate in a cell type-specific and context-dependent manner to amplify antiviral innate immunity and efficiently prevent virus spreading. The secreted enzyme ectonucleotide pyrophosphatase phosphodiesterase 1 (ENPP1) modulates this immune response by hydrolyzing extracellular 2′3′-cGAMP^36^. Importantly, transport of extracellular 2′3′-cGAMP into macrophages and other immune cells, and subsequent STING activation, enhances antitumor immunity^37,38^. Inhibitors of ENPP1 potentiate the antitumor response^39,40^.

### IFN-independent activities of mammalian STING

Although STING was initially discovered for its role in the regulation of IFN gene expression, and the IFN response is the best-understood aspect of STING signaling in mammals, IFN-independent functions are emerging for the cGAS-STING pathway. Indeed, upon activation, STING can also activate (i) the transcription factor NF-κB, leading to inflammation, (ii) autophagy, and (iii) the unfolded protein response (UPR)^14,41–44^. STING was also reported to inhibit translation^45^. Of note, the expression in mice of STING gain-of-function alleles mimicking the mutations observed in SAVI patients triggers IRF3- and IFN-independent immune cell dysregulation^46,47^. This highlights the importance of investigating the function of STING beyond regulation of IFN. As mentioned above, signaling by STING involves recruitment of the kinase TBK1 and the transcription factor IRF3 to the CTT domain of STING. Interestingly, this CTT domain is characteristic of vertebrate animals and seems to have emerged simultaneously with IFNs^13^. A comparative study of the signaling function of the CTT domain from 20 vertebrate STINGs revealed that 18 of them (the exceptions being the molecules from two *Xenopu*s species [amphibians]) mediated the activation of IRF3 and NF-κB when expressed in human cells^48^. Induction of IRF3-dependent transcription was always stronger than that of NF-κB, with the interesting difference of the molecules from salmon and zebrafish, for which induction of NF-κB was stronger than that of IRF3 by more than 100-fold. In ray-finned fishes, the CTT domain gained a sequence motif mediating the recruitment of TRAF6, resulting in rewiring of signaling and explaining the bias towards NF-κB signaling. Overall, this study revealed that (i) the CTT domain is composed of discrete modules driving different signaling outputs according to the species and (ii) the activation of IRF3 does not represent the major transcriptional output of signaling in all vertebrate species^48^.

Characterization of STING signaling revealed a key role for the phosphorylation of the serine at position 365 in the CTT domain of mouse STING in the recruitment and subsequent activation of IRF3 by TBK1. As a result, knock-in mutant mice in which this critical serine residue has been replaced by an alanine (S365A mutants) fail to induce IFN upon stimulation of the pathway. However, autophagy and NF-κB signaling can still be induced by STING in these mice, providing a powerful tool to unravel the relevance of the IFN-independent signaling arm downstream of STING *in vivo*. Strikingly, these mice were found to be resistant to infection by herpes simplex virus (HSV)-1, revealing that interferons are not absolutely required for the antiviral function of STING^49,50^. Yan and collaborators went on to study the transcriptome of bone marrow-derived macrophages (BMDMs) and T cells from wild-type and *STING^S365A^* mutant mice stimulated with DMXAA, a potent STING agonist. This revealed striking differences between the two cell types, with a largely IFN-independent STING signaling in T cells compared to BMDMs^50^. These results reveal that STING plays a broader role than previously thought based on studies that initially investigated the innate arm of immunity and focused mainly on cells of the myeloid lineage. Identification of critical residues in STING for other signaling outputs, e.g. NF-κB, autophagy, and the UPR, will allow the establishment of mouse models to investigate the contribution of these pathways to STING signaling *in vivo*, as described above for S365A and activation of IRF3. For example, STING knock-in mutant mice with the mutation L373A, which prevents the recruitment of TBK1 and the activation of both IRF3 and NF-κB, have impaired antiviral and antitumor activity, revealing the contribution of the evolutionarily conserved transcription factors NF-κB to STING function^51^. Interestingly, several recent studies have begun to shed light on the origins of cGAS-STING signaling and point to an ancestral function in connection with NF-κB signaling.

## Function and regulation of the cGAS-STING cassette in invertebrate animals

Bioinformatics analyses have revealed the presence of STING orthologs in the genome of most animals, with some intriguing exceptions, e.g. nematodes and mosquitoes^11,13^. A STING ortholog is also present in the genome of choanoflagellates, a small group of single-cell or colonial protists representing the closest living unicellular relative of animals. Remarkably, STING from animals as diverse as the worm *Capitella teleta* (Annelida), the oyster *Crassostrea gigas* (Mollusca), and the sea anemone *Nematostella vectensis* (Cnidaria) were found to be able to bind both 3′3′-CDNs and 2′3′-cGAMP. Moreover, a cGAS-like gene from *N. vectensis* encodes an enzyme producing 2′3′-cGAMP^12,14^ (Figure 2). These observations raise questions about the function of the cGAS-STING cassette in early metazoans: is it involved in antibacterial or antiviral immunity? What kind of response does it trigger in organisms devoid of IFNs?

### STING and antiviral immunity in the model insect Drosophila

Although the STING gene is absent from the genome of the nematode *Caenorhabditis elegans*, it is present in another well-characterized invertebrate model organism, the fruit fly *Drosophila melanogaster*^11^. Innate immunity in *Drosophila* involves (i) a cellular response with dedicated blood cells phagocytosing microorganisms or infected dying cells, encapsulating invading parasites, or producing melanin and toxic reactive oxygen species^52^ and (ii) a humoral response characterized by the inducible expression of a cocktail of potent antimicrobial peptides (AMPs). Surface epithelia also play an important role in the control of infections in flies^53^. Of note, induction of AMPs is regulated by two evolutionarily conserved signaling pathways, Toll and IMD, which control the activity of transcription factors of the NF-κB family, DIF and Relish, respectively. While the Toll pathway shares similarities with the IL-1R signaling pathway, the IMD pathway is evocative of the TNF receptor pathway^54^. In addition, RNAi plays a critical role in the control of viral infection. Viral double-stranded RNAs generated during replication are processed into 21-nucleotide-long small interfering (si) RNAs by the RNase III enzyme Dicer-2, and then loaded onto Argonaute-2, an RNase H-like enzyme using siRNAs as guides to target viral RNAs^55^. Viral infection in *Drosophila* is also associated with upregulation of a large number of genes, evocative of an induced response to viral infection^56^. The recent discovery of IFN-like immune responses in oysters indeed supports the existence of such a response in invertebrates^57^. The function of STING in innate immunity in *Drosophila* only recently came into the spotlight.

Noting the presence of a STING gene in flies (*dSTING*), Goodman and collaborators first reported impaired induction of the IMD pathway following infection of *dSTING* mutant flies by the intracellular bacteria *Listeria monocytogenes* and impaired resistance to the infection. No phenotype was observed in flies mutant for *CG7194*, the closest homolog to cGAS in *Drosophila*, but AMPs could be induced by the bacterial CDN 3′3′c-di-GMP. Overall, these data led the authors to propose that, in *Drosophila*, STING functions as a direct sensor of bacteria, through binding of CDNs, and activates an IMD- and Relish-dependent antibacterial response^58^. By contrast, two other studies connected STING to antiviral immunity, although through different mechanisms. Using the fly model to unravel the mechanisms of innate defense against Zika virus (ZIKV), which causes neurological complications, Cherry and colleagues observed that infection triggers the activation of the IMD pathway in the fly brain and that flies mutant for Relish or peptidoglycan recognition protein (PGRP)-LC and -LE, two PRRs activating the IMD pathway, were more susceptible to infection than controls. They further showed that STING expression was induced by ZIKV infection and that this induction depended on Relish, suggesting that STING functions as an NF-κB-regulated antiviral effector. STING was indeed found to induce autophagy in the *Drosophila* brain, which was associated with protection against ZIKV^59^. Finally, our group followed up on the observation that a number of insect DNA viruses independently hijacked a gene encoding an immunomodulatory cytokine downregulating the activation of the IMD pathway^60^. This prompted a thorough investigation of the contribution of this pathway to antiviral immunity, which revealed that two of its components, the kinase IKKβ and Relish, but not the pathway as a whole, are required in a cell line and *in vivo* to resist infection by two picorna-like viruses. Genome-wide analysis revealed that one of the genes induced by these viruses with positive-sense (+)ssRNA genomes in an IKKβ- and Relish-dependent manner was *dSTING*. Investigation of its function revealed that dSTING acts upstream of IKKβ and Relish in a pathway different from the IMD pathway and regulating the expression of a distinct set of genes^61^. More recently, we reported that injection of CDNs in flies results in the induction of STING-regulated genes (SRGs) and protection against viral infections. Importantly, this protection is completely dependent on dSTING and Relish but does not require ATG7 and AGO2, two key components of canonical autophagy and RNAi, respectively^62^. While the function of SRGs remains largely unknown, two of them, *Vago* and *Nazo*, have been shown to participate in antiviral resistance^61–63^. This study further revealed that, although 3′3′ CDNs derived from bacteria can activate dSTING, the strongest agonist is 2′3′-cGAMP, suggesting that an enzyme producing this CDN is present in flies. Interestingly, a study in another insect, the silkworm *Bombyx mori*, connected STING to Relish activation in response to infection with nucleopolyhedrovirus (NPV), a DNA virus from the *Baculoviridae* family. These authors further detected the production of cGAMP in a silkworm cell line following viral infection^64^.

Overall, these studies reveal that insect STING regulates NF-κB-dependent responses and is involved in antiviral immunity, although a participation in antibacterial immunity may also be possible^58^ (but see also^61,62^) (Figure 2). While STING may also regulate autophagy to control ZIKV in *Drosophila*, the biological significance of this finding is unclear since mosquitoes do not contain a STING gene and autophagy is proviral for ZIKV in mammalian cells^65^. The identification of genes encoding cGAMP nucleases in the genomes of Lepidopteran hosts and viruses further points to a key role of cGAS-STING signaling in antiviral immunity in insects.

### The poxin family of 2′3′-cGAMP nucleases radiated from insect viruses

As expected from the pressure the cGAS-STING pathway exerts on them, several viruses have evolved suppressor mechanisms (reviewed in 66). Among them, poxins are 2′3′-cGAMP-specific nucleases that owe their name to the family of the virus in which they were first identified, vaccinia virus (VACV, a member of the *Poxviridae*)^67^. Sometimes fused to a C-terminal domain related to Schlafen proteins, poxins prevent the induction of the STING-TBK1-IRF3 signaling axis in mammalian cells, in effect allowing the viruses to escape a potent IFN response^67,68^. Intriguingly, the closest homologs to VACV poxin are found in the genomes of insect DNA viruses of the *Alphabaculovirus* genus but also in the genome of Lepidopteran insects, which host these viruses. Importantly, these insect poxins share with their homologs from mammalian viruses the specificity for 2′3′-cGAMP and fail to cleave 3′3′-cGAMP, providing further evidence that CDNs with a 2′–5′ linkage play a significant role in antiviral immunity in insects^67^. Determination of the X-ray crystal structure of baculovirus and Lepidopteran host poxins revealed a relationship with self-cleaving viral proteases that operate to process the polyproteins translated from (+)ssRNA viruses. Based on these results, functional poxin enzymes could be identified in several insect-specific RNA viruses, some of which retained the self-cleaving protease activity^69^. The picture emerging from these data is that poxins originated from insect viral proteases, which acquired a secondary nuclease activity. These genes were subsequently endogenized in the genomes of the insect hosts for these viruses and eventually transferred to poxviruses, which have a notorious ability to acquire genes through horizontal transfer. The biochemical characterization of two viral poxins (from VACV and the baculovirus *Autographa californica* nuclear polyhedrosis virus, AcNPV) and one host-encoded poxin (from the cabbage looper *Trichoplusia ni*) pointed to interesting differences between the cellular and viral homologs. Indeed, both viral poxins display comparable high affinity for 2′3′-cGAMP compared to the *T. ni* poxin. By contrast, the host-encoded poxin tested exhibits a higher reaction rate constant than the two viral proteins^69^. Although these results will of course need to be extended to other members of the family, the differences observed suggest that viral poxins are tailored for immune evasion through efficient depletion of even low levels of 2′3′-cGAMP, whereas host poxins may function as immunomodulators, regulating the level of second messenger produced and clearing excessive amounts to avoid deleterious overactivation of the pathway^69^. The fact that host-encoded poxins appear to be upregulated by infection in two transcriptomic studies support such a role, although, here again, additional functional studies in Lepidopteran insects are warranted. In this regard, one last intriguing observation made by Kranzusch and collaborators is that many poxin genes, both viral and cellular, express isoforms with and without signal peptides for secretion^69^. Therefore, these enzymes could function in the cytosol, but also extracellularly, suggesting that the role of 2′3′-cGAMP as an immunotransmitter recently reported in mammals is evolutionarily ancient. Investigation of the function and regulation of these isoforms will clarify the role of 2′3′-cGAMP in the infected cells but also in systemic responses to viral infection in insects.

## cGAS-STING signaling originated in bacteria

As mentioned above, bacteria produce CDNs to control a diverse set of cellular responses, such as growth, osmoregulation, chemotaxis, and virulence^70^. Three distinct structural families of CDN synthases producing these CDNs have so far been identified: (i) the GGDEF c-di-GMP synthase family, (ii) the DAC/DisA c-di-AMP synthase family, and (iii) the cGAS/DncV-like nucleotidyltransferase (CD-NTase) family. Whereas the first two families encompass prokaryotic proteins, the last one—as indicated by its name—contains both prokaryotic and eukaryotic proteins, including mammalian cGAS^71^. In bacteria, CD-NTases catalyze the production of a range of cyclic dinucleotides and trinucleotides (CTNs). These results reveal the ancient origin of CD-NTases and raise the question of their function in bacteria.

### CD-NTases and antiphage immunity in bacteria

**Figure 3.**
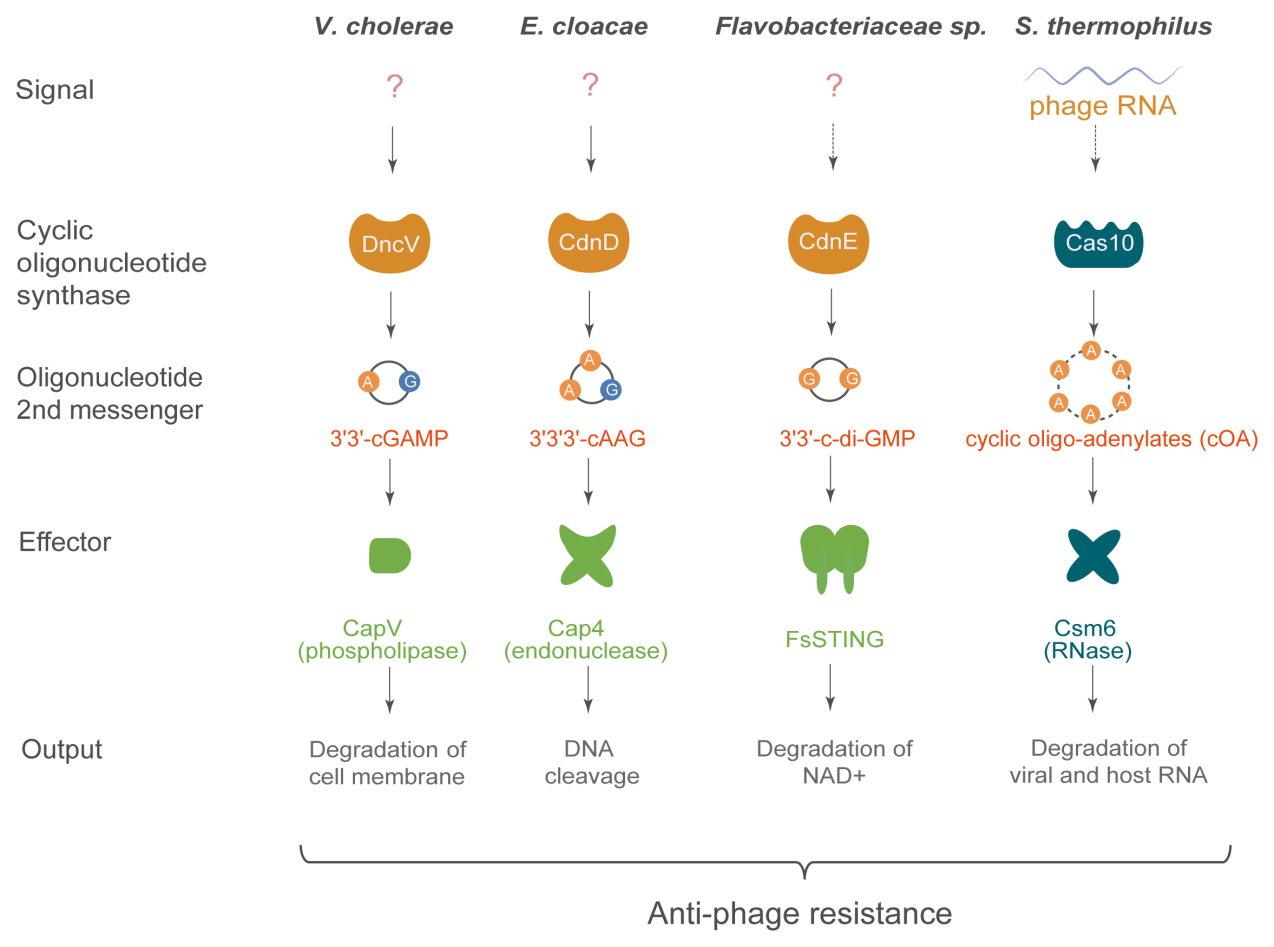
Cyclic oligonucleotide-based antiphage signaling system (CBASS) in bacteria. In bacteria, members of the cyclic guanosine monophosphate–adenosine monophosphate synthase (cGAS)/DncV-like nucleotidyltransferase (CD-NTase) family are activated by an unknown mechanism in response to phage infection and produce second messengers that activate antiviral effector mechanisms. Cas10, an enzyme of the type III CRISPR/Cas system, is structurally distinct from CD-NTases but also synthesizes cyclic oligonucleotide second messengers that activate antiphage effectors. The CARF domain binding to cyclic oligo-adenylate in Csm6 is structurally related to the SAVED domains found in effectors of CBASS, e.g. Cap4. Note that bacterial FsSTING contains a TIR domain mediating the degradation of NAD^+^ upon activation. CARF, CRISPR-associated Rossmann fold; *E. cloacae*, *Enterobacter cloacae*; NAD^+^, β-nicotinamide adenine dinucleotide; SAVED, SMODS-associated and fused to various effector domains; *S. thermophilus, Streptococcus thermophilus*; STING, stimulator of interferon genes; TIR, Toll/interleukin-1 receptor; *V. cholerae*, *Vibrio cholerae*.

One characteristic of antiphage defense systems in bacteria is that they involve genes clustered together in operons. Noticing that CD-NTases in bacteria frequently cluster with defense genes, Sorek and collaborators introduced the 4-genes operon containing the CD-NTase coding gene *DncV* from the bacteria *Vibrio cholerae* into a strain of *Escherichia coli* lacking this system and showed that it conferred resistance to an array of phages belonging to several families^72^. This protection required *DncV* and was lost when two amino acids essential for the catalytic activity of the enzyme were mutated. A second gene from the operon, encoding a patatin-like phospholipase, was found to be essential for broad defense against phages. This enzyme degrades the bacterial membrane, causing cell death and interrupting phage replication^73^. Importantly, the phospholipase can be activated *in vitro* by bacteria lysates collected after phage infection, in which 3′3′-cGAMP is detected by mass spectrometry. These results indicated that a cyclic oligonucleotide-based antiphage signaling system (CBASS) operates in bacteria to control phage infections, suggesting that the antiviral functions of cGAS and OAS in vertebrates were inherited from prokaryotes^72^. Of note, CBASS is not the only defense system producing nucleotide-based second messenger signals in bacteria. Indeed, the type III CRISPR/Cas system (a total of 6 different types of CRISPR/Cas have been identified in bacteria, each relying on a different set of Cas proteins) involves the large multidomain protein Cas10, which degrades foreign DNA but also generates 2- to 6-nucleotide-long cyclic oligo-adenylates (cOA). cOA then bind to the CRISPR-associated Rossmann fold (CARF) domains of the homodimeric enzyme Csm6, triggering its RNase activity, thus interrupting phage replication^74,75^ (Figure 3). This provides an intriguing conceptual similarity with the activation of RNase L by OAS-produced linear oligoadenylates in mammalian antiviral immunity.

### Some bacterial CD-NTases produce 2′-5′ linked cyclic oligonucleotides to restrict phage infection

Noting that the CBASS operons encoding bacterial CD-NTases often do not contain patatin-like phospholipases, Kranzusch and collaborators set out to identify other bacterial effectors regulated by CDNs or CTNs. This led to the identification of more than 2,000 effectors, including nucleases, proteases, β-nicotinamide adenine dinucleotide (NAD^+^) hydrolases, and putative pore-forming proteins^76^. All of these proteins, present in about one-third of sequenced operons containing a CD-NTase, share a SMODS-associated and fused to various effector domains (SAVED) domain, which accommodates the nucleotide second messenger. Unexpectedly, solving the structure of the SAVED domain unveiled two fused CARF domains, revealing convergence between CBASS and type III CRISPR/Cas systems for the sensing of cyclic oligonucleotide second messengers. The potential for cross-talk between the two antiphage defense systems is reinforced by the observation that some type III CRISPR/Cas operons contain genes encoding SAVED domains^76,77^. Of note, as mentioned above for Csm6, activation of type III CRISPR involves homodimeric CARF domain-containing proteins, which change conformation upon binding to a ligand with twofold symmetry. Fusion of two CARF domains into a SAVED domain resulted in a domain able to accommodate a wide variety of cyclic oligonucleotide second messengers, including asymmetric ones. Indeed, the CD-NTase from *Acinetobacter baumannii* synthesized the CTN 2′3′3′-cAAA, demonstrating that 2′–5′ phosphodiester linkages, although rare, are also present in bacteria, where they participate in antiphage defense^76^. In summary, a large array of distinct oligonucleotides with different nucleobase composition, ring size, and 2′–5′ or 3′–5′ linkage, synthesized by a diversity of CD-NTase enzymes, activate a panel of different antiphage effector proteins in bacteria (Figure 3). This diversity probably offers an efficient safeguard against phage replication.

### Bacterial STING proteins function as CDN-regulated antiphage effectors

Interestingly, bioinformatic analysis of phage defense operons revealed the presence of genes encoding proteins with predicted homology to STING^72,78,79^. Structural analysis revealed that these proteins indeed share the overall architecture of metazoan STING in spite of a 20% smaller size and more compact organization. Notably, bacterial STING binds to c-di-GMP and binds poorly or not at all to CDNs containing a 2′–5′ linkage, including 2′3′-cGAMP^78^. Only a few bacterial STING proteins contain predicted transmembrane helices but most of them also contain a Toll/interleukin-1 (IL-1) receptor (TIR) domain. TIR domains are present in plant and animal proteins involved in host defense. While the TIR domain of Toll and IL-1 receptor families function as homotypic protein–protein interaction domains to recruit signaling adaptors, some TIR domains in bacteria, plants, and animals have catalytic activity and degrade NAD^+^, which is essential for cellular metabolism^80^ Indeed, activation of a bacterial TIR-STING protein with c-di-GMP resulted in rapid and efficient hydrolysis of NAD^+^. Interestingly, this activation was accompanied by assembly of the TIR-STING proteins into filaments, as previously reported for human STING, which oligomerizes upon binding 2′3′-cGAMP, thus leading to TBK1 activation^78,81^.

In summary, both CD-NTase enzymes producing CDN signals and STING-like molecules acting as a receptor for this signal were already present and active in defense against viruses in prokaryotic cells (Figure 3). 2′–5′ phosphodiester linkage in the CDNs is also present in some bacteria, although not in connection to STING activation. Overall, these results suggest that the eukaryotic ancestors of animals inherited each component of the cGAS-STING signaling pathway from their prokaryotic precursors, a hypothesis supported by the identification of STING-TIR fusion proteins in some metazoans, e.g. oysters^78^.

## Concluding remarks

The field of cGAS-STING signaling has seen a number of significant new developments in recent years. Not least among them, we now have a clear idea of the origin of the molecules composing the cGAS-STING signaling cassette, which arose in prokaryotes, where they participate in defense against bacteriophages. STING signaling is also emerging as an important antiviral mechanism in insects, suggesting a striking structural and functional conservation of this cassette during evolution to restrict viruses, a universal threat for cellular life. Argonaute proteins represent another example of ancient and conserved factors participating in antiviral restriction in all domains of life^82^. One intriguing difference between cGAS-STING signaling in mammals and that in bacteria is the use of a mix of 2′–5′ and 3′–5′ linkages in the products of cGAS and OAS, whereas in the vast majority of cases bacteria favor a 3′–5′ linkage. It is now clear that these differences can have important consequences for both activation of downstream effectors and degradation by nucleases^36,69^. Mammalian and insect STING detect both 2′3′- and 3′3′-CDNs but appear to favor 2′3′-cGAMP as a ligand^9,62^, and the cGAS enzyme from the sea anemone produces 2’3’-cGAMP^14^, raising the possibility that the 2′–5′ linkage was co-opted in metazoans to increase the signal-to-noise ratio in the context of viral infections in multicellular organisms tightly associated with a microbial flora. The identification and characterization of additional cGAS enzymes in invertebrates will undoubtedly clarify this issue.

As for all active fields of research, the recent insights on cGAS-STING signaling raise new fascinating questions. Regarding the evolution of the pathway, one of them is the regulation of the activation of CD-NTases in bacteria, which seem to be constitutively active *in vitro*^71,72,76^. Do CD-NTases respond to changes in the cell (e.g. altered metabolism and modification of HORMA-domain proteins^83^) or can they be activated upon sensing viral components like PRRs in animals and as shown for Cas10 (a GGDEF-type synthase), which is activated by phage RNA in *Streptococcus thermophilus*^74,75^? Similarly, how is the STING pathway activated in invertebrate animals? It has been noted that the zinc ribbon present in mammalian cGAS and important for DNA sensing is absent from cGAS-like molecules in invertebrates^13^. This suggests that these candidate PRRs may respond to another type of nucleic acid, a hypothesis consistent with the fact that STING is activated by RNA viruses in flies^59,61^. Another question raised by the numerous different effector molecules activated by CDNs and CTNs in bacteria is whether CDNs can activate receptors other than STING in animals. The only such alternative receptor reported today, the mouse oxidoreductase RECON, can sense bacterial CDNs and CTNs and controls NF-κB activity^71,84,85^.

To conclude, it is remarkable that, in parallel to the significant progress made during the past 2 years on the understanding of the evolutionary history of cGAS/STING signaling, exciting new developments occurred in mammals, testifying to the liveliness of a field where much remains to be learned. One consequence of the emerging conservation of the pathway in invertebrates is that model organisms like *Drosophila* may provide some insights on still-mysterious facets of cGAS/STING biology. Elucidation of the mechanism through which STING activates IKKβ and Relish in *Drosophila* can, for example, be expected to shed light on the still-elusive activation of NF-κB by STING in mammals. The fact that Lepidopteran insects appear to secrete poxins^62^ and that injection of 2′3′-cGAMP in the body cavity of flies results in strong antiviral protection^62^ argue that the function of this CDN as an immunotransmitter is evolutionarily ancient and could be studied using the genetic resources of the *Drosophila* model. Finally, it will be interesting to see where the invertebrate cGAS enzymes reside. Should they also be located in the nucleus, model organisms like *Drosophila* will be ideally suited to decipher their role and regulation in this critical cellular compartment.
